# Defining microbial biomarkers for risk of preterm labor

**DOI:** 10.1007/s42770-019-00118-x

**Published:** 2019-07-22

**Authors:** Anderson Santos de Freitas, Priscila Caroline Thiago Dobbler, Volker Mai, Renato S Procianoy, Rita C. Silveira, Andréa Lúcia Corso, Luiz Fernando Wurdig Roesch

**Affiliations:** 1 Centro Interdisciplinar de Pesquisas em Biotecnologia – CIP-Biotec, Campus São Gabriel, Universidade Federal do Pampa, São Gabriel, Rio Grande do Sul Brazil; 2 Department of Epidemiology, College of Public Health and Health Professions and College of Medicine, Emerging Pathogens Institute, University of Florida, Gainesville, FL 32611 USA; 3 Serviço de Neonatologia do Hospital de Clínicas de Porto Alegre, Universidade Federal do Rio Grande do Sul, Porto Alegre, Rio Grande do Sul Brazil

**Keywords:** Next-generation sequencing, Vaginal microbiome, *Lactobacillus*, Brazilian microbiome

## Abstract

Preterm birth remains the main contributor to early childhood mortality. The vaginal environment, including microbiota composition, might contribute to the risk of preterm delivery. Alterations in the vaginal microbial community structure might represent a risk factor for preterm birth. Here, we aimed to (a) investigate the association between preterm birth and the vaginal microbial community and (b) identify microbial biomarkers for risk of preterm birth. Microbial DNA was isolated from vaginal swabs in a cohort of 69 women enrolled at hospital admission for their delivery. Microbiota was analyzed by high-throughput 16S rRNA sequencing. While no differences in microbial diversity measures appeared associated with the spontaneous preterm and full-term outcomes, the microbial composition was distinct for these groups. Differential abundance analysis showed *Lactobacillus* species to be associated with full-term birth whereas an unknown *Prevotella* species was more abundant in the spontaneous preterm group. Although we studied a very miscegenated population from Brazil, our findings were similar to evidence pointed by other studies in different countries. The role of *Lactobacillus* species as a protector in the vaginal microbiome is demonstrated to be also a protector of spontaneous preterm outcome whereas the presence of pathogenic species, such as *Prevotella* spp., is endorsed as a factor of risk for spontaneous preterm delivery.

## Introduction

According to the World Health Organization, every birth before 37 weeks of pregnancy is considered preterm. Each year, about 15 million babies are born prematurely in the world [1]. Prematurity is the leading cause of mortality before 4 weeks of life and the second until 5 years of age [1, 2]. Preterm birth also leads to disorders related to brain development, the deficit of attention, hyperactivity [3–5], autism [6], and respiratory problems [7]. In the USA, the preterm delivery rate is around 9.6% [8] while in Europe and other developed countries it is between 5 and 9% [9]. In developing countries, especially South Asia and sub-Saharan Africa, the preterm birth rates are above 15% [1].

The epidemiological and clinical natures of preterm birth are not yet fully understood [2, 10, 11]. Nevertheless, preterm delivery is associated with type 2 diabetes [12], weight gain, chronic postpartum hypertension [13], air pollution [14], psychological and social conditions, physical exertion during pregnancy [15], diet, hygiene, and access to health care [2, 11]. Several studies attempted to map the endemicity of this disease and their results indicate a higher incidence in black women, in women under low socioeconomic levels, in smokers, in pregnancies of twins, and/or more advanced age [1, 2, 11, 16–20].

Different microbes also have been correlated with preterm delivery [21, 22], but microbial community-level studies represent a suitable and fast alternative to better understand the relationship between the microbial community and the preterm birth. As women from different ethnic backgrounds have different vaginal microbial communities [23–25], local attempts to detect and associate microbial communities with preterm birth are required [26]. Such regional attempts might sum up with other worldwide initiatives to elaborate a prediction risk assessment plan based on the vaginal microbial community. Within this work we aimed to (a) investigate the association between preterm birth and the vaginal microbial community and (b) identify microbial biomarkers for risk of preterm birth.

## Material and methods

### Experimental design

This study was carried out with samples collected from women attending the Hospital de Clínicas de Porto Alegre (HCPA). Experimentation used a convenience sampling strategy. Expecting mothers were enrolled at hospital admission for their delivery between May 2014 and March 2016. All women provided written informed consent to allow their samples to be used in the study. The ethics committee of HCPA approved the study protocol. Exclusion criteria were: (1) HIV or congenital infections,( 2) drug user or alcoholic, (3) urinary tract infections or (4) antibiotic usage in the third trimester of gestation, (5) urogenital infection in the last 3 months, and (6) gestational diabetes.

Sixty-nine pregnant women were analyzed in this study. Twenty-three of them had spontaneous preterm labor (before 33 weeks of gestation), whereas 29 had spontaneous term labor. Another 17 women had non-spontaneous labor but, due to medical reasons affecting pregnancy, had cesarean delivery before 33 gestational weeks. Those subjects called hereinafter “non-spontaneous preterm” group, were used as a second control because they present a microbial community that might not be associated with spontaneous preterm delivery but have a better match in terms of gestational age with the spontaneous preterm group. All pregnant women sampled on this work had vaginal swab (Sterile Specimen Collection Swabs to collect specimens from soft tissue surfaces-Labor swab®) collected up to 4 hours before labor begins, as described by Roesch and colleagues [27]. Collected swab samples were stored at − 80 °C until DNA extraction. The characteristics from the mothers enrolled in this experiment include maternal age, previous pregnancies, gestational age, incidence of chorioamnionitis, preeclampsia, infection by Group B *Streptococcus*, intrapartum penicillin administration, and delivery mode.

### Microbial DNA extraction, 16S rRNA amplification and sequencing, and data processing

Microbial DNA was extracted from frozen swab samples as previously described by Roesch et al. [27]. All DNA samples were kept at − 20 °C until use in PCR reactions. Vaginal microbiota was determined by amplification of the V4 region of the 16S rRNA gene and downstream sequencing on the Ion PGM Platform (Thermo Fisher Scientific, Waltham, MA, USA) with the bacterial/archaeal primers 515F and 806R [28]. All samples were PCR-amplified using barcoded primers linked with the Ion adapter “A” sequence (5′-CCATCTCATCCCTGCGTGTCTCCGACTCAG-3′) and Ion adapter “P1” sequence (5′-CCTCTCTATGGGCAGTCGGTGAT-3′) to obtain a sequence of primer composed by A-barcode-806R and P1-515F adapter and primers. PCRs were carried out in 25 μL reactions contained 2 U of Platinum® Taq DNA High Fidelity Polymerase (Invitrogen, Carlsbad, CA, USA), 4 μL 10X High Fidelity PCR Buffer, 2 mM MgSO_4_, 0.2 mM dNTP’s, 0.1 μM of both the 806R barcoded primer and the 515F primer, 25 μg of Ultrapure BSA (Invitrogen, Carlsbad, CA, United States) and approximately 50 ng of DNA template. PCR conditions used were: 95 °C for 5 min; 30 cycles of 94 °C per 45 s denaturation, 56 °C per 45 s annealing, and 72 °C per 1 min extension; followed by 72 °C per 10 min for final extension. Fragments presenting around 400 base pairs from resulting PCR products were purified with the Agencourt® AMPure® XP Reagent (Beckman Coulter, Brea, CA, USA), and final concentration of the PCR products was quantified by using the Qubit Fluorometer kit (Invitrogen, Carlsbad, CA, United States) following manufacturer’s instructions. Finally, reactions were combined in equimolar concentrations to create a mixture composed of amplicon fragments of each sample. This composite sample was used for library preparation with Ion OneTouch™ 2 System with the Ion PGM™ Template OT2 400 Kit Template (Thermo Fisher Scientific, Waltham, MA, USA). The sequencing was performed using Ion PGM™ Sequencing 400 on Ion PGM™ System using Ion 318™ Chip v2 with a maximum of 40 samples per microchip.

Raw reads were analyzed according to the pipeline proposed by the Brazilian Microbiome Project [29]. A table of operational taxonomic units (OTUs) was constructed by using the UPARSE pipeline [30], with a minimum similarity cutoff value of 97% for clustering and a maximum expected error of 0.5%. Taxonomic classifications were made on QIIME 1.9.0 [31], based on UCLUST method against the SILVA ribosomal RNA gene database version 128[32] with a confidence interval of 95%. Sampling effort was measured by the Good’s coverage [33].

### Data analyses

Maternal variables were analyzed into the R environment [34]. Numeric variables were summarized as average ± SEM and compared using the Kruskal-Wallis followed by a post hoc Dunn test. Categorical variables were compared using chi square post hoc test.

The 16S rRNA database was analyzed through the phyloseq [35] and the Microbiome [36] packages after removing singletons and rarefying the dataset to the minimum library size. Possible confounding variables were tested by permutational multivariate analysis of variance (PERMANOVA) into the vegan package [37].

Initial insights about general microbial structure were provided by analyses of relative abundance (measured by Kruskall-Wallis post hoc Dunn test) of the most frequent genera and alpha diversity tests.

The differential abundance analysis, applied to find microbial biomarkers of preterm birth, was performed by using the DESeq2 [38] with the raw (non-rarefied) dataset. Briefly, after removing the samples from mothers treated with intrapartum penicillin from the dataset, the OTU table was conglomerated at species level. Taxa not seen more than 3 times in at least 20% of the samples were removed and the number of sequences per OTU was transformed by calculating the geometric mean. Two different contrasts were applied: (a) term birth versus preterm birth and (b) false-preterm birth versus preterm birth. The FDR method was used to control for false discovery rate. Additional correlation analysis between OTUs was tested by using SparCC approach [39].

## Results

### Maternal variables used for comparison between groups

The characteristics of the three groups are shown in Table 1. Maternal age ranged from 23 to 30 years but was not significantly different between women with subsequent term or preterm labor. Nonetheless, women significantly older composed the non-spontaneous preterm group. The number of previous pregnancies was also similar in all, spontaneous term labor and spontaneous preterm labor groups as well as in non-spontaneous preterm group. Moreover, as expected, gestational age was significantly different between term and preterm groups, but not different between spontaneous preterm and non-spontaneous preterm groups. Women who had preterm labor presented a higher incidence of chorioamnionitis than the term group. This condition did not differ between term and non-spontaneous preterm groups. Only the preterm and the non-spontaneous preterm groups presented cases of infection by Group B *Streptococcus* (GBS). Although the GBS infection rate was higher in the non-spontaneous preterm group, the incidence of GBS infection was not statistically different between spontaneous preterm and non-spontaneous preterm groups. Most cases of non-spontaneous preterm labor presented preeclampsia, whereas there were no cases in the term group and only three cases in the spontaneous preterm labor group. The intrapartum penicillin was used in 15 preterm cases. The term and non-spontaneous preterm groups did not receive prophylactic antibiotics. Intravenous penicillin was administered approximately 4 h before labor in many preterm samples due to a positive test for GBS or due a suspicion of infection in absence of a test. Finally, the three groups differed in terms of vaginal or cesarean delivery. The preterm condition was the major driver of cesarean, especially in the non-spontaneous preterm labor group.

**Table 1:** Maternal variables used for comparison between groups

Variables	Spontaneous term labor (*n* = 29)	Spontaneous preterm labor (*n* = 23)	Non-spontaneous preterm labor (*n* = 17)
Maternal age (years)	25.03 (± 1.13)^a^	23.60 (± 1.28)^a^	30.24 (± 1.75)^b^
Previous pregnancies	2.00 (± 0.17)^a^	1.87 (± 0.34)^a^	2.12 (± 0.28)^a^
Gestational age (weeks)	39.60 (± 0.20)^a^	30.70 (± 0.39)^b^	29.42 (± 0.59)^b^
Chorioamnionitis	0^a^	8^b^	2^b^
Preeclampsia	0^a^	3^a^	14^b^
GBS* infection	0^a^	5^b^	4^b^
Intrapartum penicillin	0^a^	15^b^	0^a^
Delivery mode (cesarean)	1^a^	10^b^	17^c^

*GBS, Group B *Streptococcus.* Numeric variables were summarized as average ± SEM and compared using the Kruskal-Wallis followed by a post hoc Dunn test. Categorical variables were compared using chi square post hoc test. Data followed by the same letter in the line represent groups without significant statistical difference (*p* > 0.05) whereas data followed by different letters in the line represent statistically different groups at the significance level of 95% (*p* ≤ 0.05)

### Controlling for confounding variables

Permutational analysis of variance was applied to test the effect of confounding variables on the microbiota analyses (Table 2). As significant reduction in taxonomic diversity of vaginal microbial community was already observed as pregnancy advances [40], we first attempted to verify the influence of gestational age on the vaginal microbiota. A pairwise analysis among the spontaneous term labor, spontaneous preterm labor, and non-spontaneous preterm labor groups revealed undetectable microbial community differences among those groups in our dataset (Table 2). In all comparisons, *R*^2^ was smaller than 1% and the *p* value was greater than 0.05. Fifteen out of 23 women from the spontaneous preterm labor group received prophylactic antibiotics during labor, whereas no women from the term group received antibiotics. The *R*^2^ for antibiotic usage was 0.034 and the *p* value was 0.043 indicating that about 3.4% of the variation in the microbial community between groups was explained by the prophylactic use of antibiotics during labor. Intrapartum antibiotics were administrated only in cases with preterm labor. For this reason, this factor could not be used in a multi-factor design with interactions. All OTUs under intrapartum antibiotics influence were removed from the dataset prior to diversity and differential abundance analysis.

**Table 2:** Permutational analysis of variance (PERMANOVA) of the Bray-Curtis dissimilarities for bacterial OTU community structure used for detection of possible confounding variables associated with preterm labor

Confounding variables	*R*^2^	*p* value
Mother’s age	0.029	0.482
Preview pregnancies	0.014	0.748
Gestational age	0.017	0.540
Corioamniotitis	0.011	0.895
Preeclampsia	0.030	0.106
GBS infection	0.013	0.734
Intrapartum penicillin	0.034	*0.043*
Delivery mode (cesarean/vaginal)	0.014	0.711

Significant value is set in italics. *p* values are based on 999 permutations

### Different microbial community structure but similar vaginal microbial diversity within treatments

**Fig. 1:**
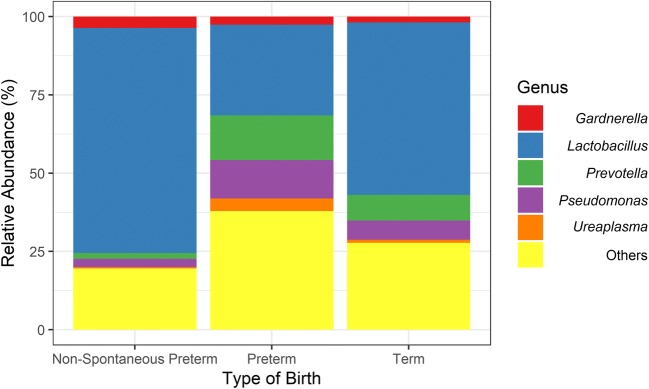
Percentage of the five most abundant microbial genera found in the three tested groups. The genus *Lactobacillus* appeared with a low mean in preterm group when compared with term (Kruskal-Wallis post hoc Dunn test, *p* = 0.003) and with marginally low mean compared with non-spontaneous preterm (*p* = 0.080). Although *Prevotella* tended to be in high abundance in preterm samples, the test only found such difference when comparing preterm with non-spontaneous preterm group (*p* = 0.016). *Pseudomonas*, *Ureaplasma*, and *Gardnerella* did not present significant difference neither sample-to-sample or among samples (*p* > 0.05)

**Fig. 2:**
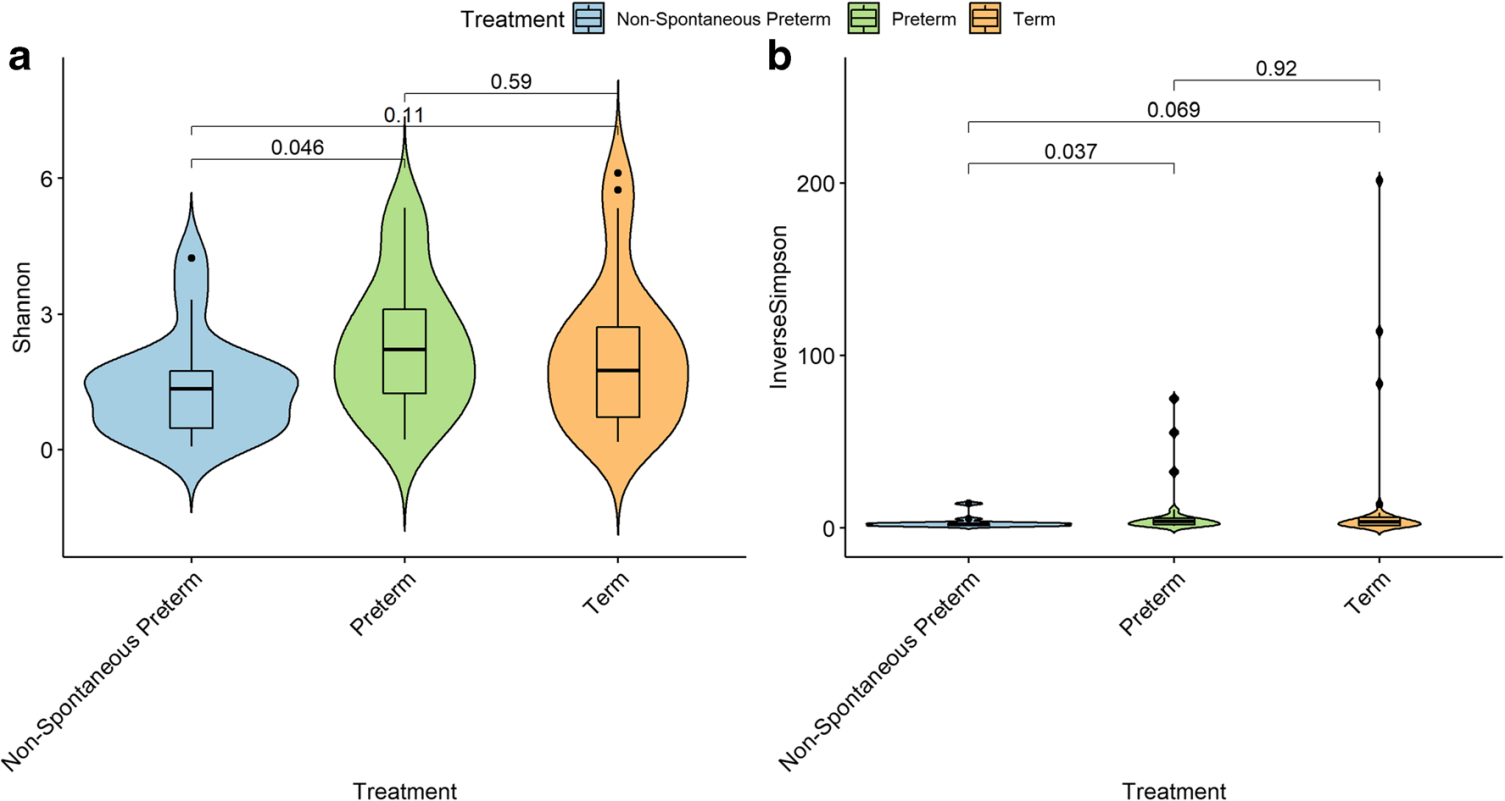
Alpha diversity measurements of microbial communities in the spontaneous preterm labor and control groups. **a** Shannon diversity index. **b** Inverse of Simpson index. Boxes span the first to third quartiles; the horizontal line inside the boxes represents the median. Whiskers extending vertically from the boxes indicate variability outside the upper and lower quartiles, and the single circles indicate outliers. No significant difference was found among the three groups (*p* > 0.05) according to the non-parametric Wilcoxon test

The mean of organisms’ abundance was particularly similar between term and non-spontaneous preterm groups as indicated by the Kruskal-Wallis post hoc Dunn test. On the other hand, preterm group presented a low mean of OTUs closest related to the genus *Lactobacillus* among its samples jointly with a tendency to a high mean of OTUs with best hit to *Prevotella* and *Pseudomonas* when compared with terms and non-spontaneous preterm (Fig. 1). Overall, the alpha diversity was low among all samples. The non-parametric Wilcox test indicated no differences in microbial diversity among the three groups tested using either Shannon or inverse of Simpson diversity indexes (Fig. 2).

### Defining the main biomarkers associated with term and preterm delivery

To outline the main microbes associated with the term and preterm labor we performed a differential abundance analysis. Pairwise comparisons among spontaneous term and spontaneous preterm groups revealed the abundance of OTUs closest related to two species of *Lactobacillus* associated with the term birth (Table 3). They were *Lactobacillus iners* and *Lactobacillus jensenii*. An unknown species related to the *Prevotella* genus was more abundant in the spontaneous preterm group. Similar tendency was also observed when comparing non-spontaneous preterm labor and spontaneous preterm labor groups. An OTU with the best hit to *Lactobacillus jensenii* was associated with subjects with non-spontaneous preterm labor while two taxa related to the *Prevotella* genus were associated with the spontaneous preterm labor. In an attempt to verify whether *Prevotella* was associated with *Lactobacillus*, we perform a correlation analysis at the genus level by using the SparCC approach [39]. No significant strong correlation (correlation coefficient = − 0.17 and *p* = 0.09) was found involving either *Lactobacillus* or *Prevotella*.

**Table 3:** Differential abundance analysis depicting vaginal microbial biomarkers associated with term or preterm labor

Base mean	Log_2_-fold change	lfcSE	Stat	*p* value	*p* adj	Closest microbial relative	Increased in
Spontaneous term versus spontaneous preterm							
22769	3.48	1.37	2.54	0.011	0.016	*Lactobacillus iners*	Spontaneous term labor
229	7.84	2.32	3.39	0.001	0.030	*Lactobacillus jensenii*	Spontaneous term labor
106	4.25	1.30	− 3.27	0.001	0.031	*Prevotella* sp.	Spontaneous preterm labor
Spontaneous preterm versus non-spontaneous preterm							
229	7.40	2.46	3.00	0.003	0.045	*Lactobacillus jensenii*	Non-spontaneous preterm labor
218	2.90	1.48	− 1.96	0.050	0.353	*Prevotella bivia*	Spontaneous preterm labor
106	5.03	1.39	− 3.61	0.000	0.026	*Prevotella* sp.	Spontaneous preterm labor

*Base mean*, the average of the normalized counts taken over all samples; *log*_*2*_*-fold change*, log_2_ fold change between the groups; lfcSE, standard error of the log_2_*-*fold change; Stat, Wald statistic; *p* value; Wald test *p* value; *p* adj, FDR-adjusted *p* value

The results indicated that the absence of high numbers of OTUs classified as *Lactobacillus*, particularly as *Lactobacillus iners* and *Lactobacillus jensenii*, might be the main difference between the vaginal microbial community of pregnant women following term or spontaneous preterm labor.

## Discussion

In this work, we attempted to detect biomarkers for preterm labor on the vaginal microbiota of pregnant women. Several studies have described the vaginal microbiota of pregnant women; however, most of them were based in the USA, Canada, Europe, or Mexico [23–25, 41–43] and came to very incongruent results. A North American NGS-based study performed by Romero and colleagues, for example, concluded that there was no difference between abundance and structure of the vaginal microbiome, independent of the type of birth [23]. On the other hand, the efforts by Hyman and collaborators, whom worked with chain-termination sequencing, conclude that mothers whom deliver prematurely present a high diverse vaginal microbiota [42]. In addition, a Canadian study by Freitas et al. not only correlates high diversity on vaginal microbiota to preterm delivery but also the presence of Mollicutes [43].

Here, we used next-generation sequencing to analyze a Brazilian cohort composed of 69 pregnant women. The unique feature of this work is the high miscegenation rates of the Brazilian population. The aforementioned studies suggest that women from different ethnic backgrounds have different vaginal microbial communities. Therefore, investigations using cohorts with different ethnic backgrounds are important to better understand the etiology of preterm labor and its relationship with microbes.

Vaginal microorganisms possess a known key role in states of health and disease acting as both generators/stimulators and protectors from diseases [24, 44, 45]. Interactions between the microbiota and human diseases occur in a two-way process. Bacteria can cause diseases as much as states of diseases can cause changes in the normal microbiota. An example is the increase in bacterial pathogen abundance in cases of depression. Gut microbes can produce identical hormones and neurotransmitters produced by humans. In turn, the bacterial receptors for these hormones influence microbial growth [46].

In this context, we presented multiple lines that lead to the presence of different vaginal microbial communities associated with the full-term and spontaneous preterm labor. The first evidence was provided by overall abundance analysis (Fig. 1). Preterm group represented differences when compared with other ones, mainly related to the decrease of general abundance of *Lactobacillus*. The second and most strong evidence was obtained by a differential abundance analysis (Table 3). Lower numbers of OTUs with best hit to species from the genus *Lactobacillus* were associated with the spontaneous preterm labor while vaginal bacterial communities rich in these microbial species (e.g., spontaneous term and non-spontaneous preterm groups) were associated with the full-term outcome. Non-spontaneous preterm labor presented similar microbial communities composition to those subjects with spontaneous term labor. Indeed, the healthy vaginal microbiota in the Brazilian pregnant woman has low microbial diversity and is dominated by *Lactobacillus* species [27]. Besides, *Lactobacillus* species are very often correlated to states of health in the vaginal environment [24, 47–50]. Bacteria from this genus present a fermentative metabolism with lactate and usually acetate, ethanol, CO_2_, formate, or succinate as products [51]. These compounds acts lowering the vaginal pH to levels around 4,5 and creating an inhospitable environment for most of pathogenic species [49, 52, 53].

On the other hand, we were able to detect the presence of OTUs closest related to the genus *Prevotella* in association with the spontaneous preterm labor. In fact, many microorganisms, just like *Prevotella* species, can produce pro-inflammatory substances that can also lead to a preterm birth [54]. Studies point to adaptation of specific *Prevotella* strains at different niches. The report by Gupta and colleagues, for example, showed 83% of the *Prevotella* genome may contribute to singletons and flexible sequences and this condition performs a key role in the adaptation to many body sites [55]. Indeed, several works indicate *Prevotella* strains related dysbiosis in states of disease in highly different body parts, i.e., asthma and bacterial vaginosis [56, 57]. *Prevotella* is still correlated with inflammatory processes by the activation of Toll-like receptor 2, which leads to production of T helper type 17 cells (Th17) and increase of interleukin 8 (IL-8), interleukin 6 (IL-6), and chemokine (C-C motif) ligand 20 (CCL20) [58]. In addition, the intrauterine infection, which may have originated in the vaginal cavity, might account for 25–40% of preterm births [59]. The most commonly associated bacteria are bacteria from the class *Mollicutes* (*Ureaplasma* species, *Mycoplasma genitalium*, and *M. hominis*, for example) [60, 61], but many other microbial species have been identified in cases of bacterial vaginosis, including *Prevotella* [62–64]. Those microbes might invade the uterus by migrating from the passage through the cervix from the vagina and infect the amniotic fluid [59]. The metabolism of some of these bacteria may also produce urease, an enzyme that catalyzes the hydrolysis of urea into carbon dioxide and ammonia. Its activity increases the vaginal pH, a stress environmental condition for the mother and the fetus that may influence in a spontaneous premature outcome [65, 66].

Callahan et al. [67] recently presented similar results. The authors studied two cohorts from different locations of the USA. A lower abundance of *L*. *crispatus* was significantly associated with the preterm birth in both cohorts. In line with our results, the cohort from Birmingham, AL, presented decreased abundance of *L*. *jensenii* associated with the preterm birth. But contrary to our results, no significant association was detected for *L*. *iners*. Moreover, among the women with lower levels of *Lactobacillus*, a higher abundance of *Gardnerella* and *Ureaplasma* was associated with the increased risk of preterm labor. According to Baldwin et al. [68], *Lactobacillus* spp. were markedly decreased when compared with vaginal swabs collected from uncomplicated pregnancy subjects with a matched gestational time. As observed in our dataset, the authors also observed deficiency of *Lactobacillus* and persistence of known pathogenic species, such as *Prevotella* sp., as a risk factor for preterm birth.

In short, reports from the aforementioned studies as well as from this one converge to a pattern of bacteria either pathogenic or related to stress conditions as increased in preterm cases. Considering this fact and the niche adaptation performed by *Prevotella* spp. [55], we are able to suggest *Prevotella* as a microbial biomarker for preterm labor in the vaginal microbiota.

## Conclusion

The relationship between the vaginal microbes and the spontaneous preterm labor was already described in racially distinct cohorts. In spite of this, to the best of our knowledge, this is the first study to describe and correlate the vaginal microbiota with the spontaneous preterm labor in a Brazilian cohort. This is especially important because: (i) preterm-microbiota associations are population dependent [67] and (ii) the Brazilian population presents high rates of miscegenation. As so, this population cannot be classified using standard stratifications of Caucasian/white and black/African American. Our results add to the ecological theory of the protective effect of *Lactobacillus* and the occurrence of other pathogenic taxa (e.g., *Prevotella*) as a possible risk factor for preterm labor.
